# Reference Material Production and Milk Protein Concentration as Elements to Improve Bluetongue Serological Diagnosis in Bulk Tank Milk

**DOI:** 10.3390/v16060915

**Published:** 2024-06-04

**Authors:** David Romero-Trancón, Marta Valero-Lorenzo, Montserrat Agüero, Rubén Villalba

**Affiliations:** Laboratorio Central de Veterinaria (LCV), Ministry of Agriculture, Fisheries and Food, Ctra. M-106, Km 1,4, 28110 Algete, Spain; bec_algete02@mapa.es (D.R.-T.); mvalero@mapa.es (M.V.-L.); maguerog@mapa.es (M.A.)

**Keywords:** bluetongue, bulk tank milk serology, surveillance, ELISA, milk protein concentration

## Abstract

The serological surveillance of bluetongue in bulk tank milk is an efficient and cost-effective method for the early detection of bluetongue virus incursions in unvaccinated free areas of the disease. In addition, the availability of standardized and reliable reagents and refined diagnostic procedures with high sensitivity and specificity are essential for surveillance purposes. However, no available reference materials for bluetongue virus serological surveillance in bulk tank milk exist. This study shows the production and characterization of reference material for the implementation of a commercially available bluetongue milk ELISA test in official laboratories, as well as the evaluation of a procedure to increase the sensitivity in samples with low levels of antibodies. This procedure, based on milk protein concentration, allowed us to notably increase the ELISA test’s analytical sensitivity, which is useful for milk samples from farms with low within-herd prevalence or pools of bulk tank milk samples. The standardized milk reference material produced here, together with the evaluated procedure to improve analytical sensitivity, could be applied as tools to ensure an accurate diagnosis by official laboratories in bluetongue unvaccinated free areas.

## 1. Introduction

Bluetongue (BT) is an infectious, non-contagious, vector-borne viral disease transmitted by *Culicoides* spp. midges that affects domestic and wild ruminants. The etiological agent, bluetongue virus (BTV), belongs to the *Orbivirus* genus and *Sedoreoviridae* family, with at least 29 antigenically distinct serotypes currently recognized [1,2,3], of which 1–24 are typical serotypes officially notifiable in the European Union [4]. Bluetongue infection can cause disease in livestock (sheep, goats, and cattle), including hemorrhagic fever, production losses, and death [5,6]. Bluetongue outbreaks cause severe economic losses due to the direct effects of the disease, trade restrictions, and the costs of surveillance and control measures [7,8]. Because of its economic impact, BT is a World Organization for Animal Health (WOAH)-listed multispecies disease and subjected to compulsory notification [9].

The worldwide distribution of BTV is mainly determined by the distribution of different *Culicoides* species [2]. Climatic change also favors the spread of midges in new geographical areas, resulting in the increase of BTV incidence and transmission [10]. Although BTV was originally restricted to Africa, since 1950, the disease has been reported in all continents except Antarctica, being recognized as an emerging transcontinental disease [2]. Since 1998, BTV has been circulating in southern European countries and over the Mediterranean basin, from which it has gradually spread to previously BTV-free areas in central and northern Europe [11,12,13]. In 2006, the incursion of a highly virulent strain of BTV-8 caused a severe epidemic in northern Europe with the spread to southern European countries in the following years [12,14]. Afterward, several European areas also suffered BTV emergence, re-emergence, or new incursions of other typical serotypes (BTV-4 in Balkans, 2014 and 2020; BTV-6 in Germany and the Netherlands, 2006; BTV-11 in Belgium, 2008; BTV-14 in Poland, 2012) [15,16,17,18]. Recently, BTV-3 has emerged in the Netherlands affecting neighboring countries (Germany, Belgium, and United Kingdom) [19,20] and threatening BTV-free areas in central and northern Europe [21].

An accurate surveillance program and early diagnosis are essential to implement control measures such as vaccination and restricting movements of viremic animals between BT-affected and BTV-free areas [6,12,22]. For these purposes, clinical samples, mostly blood from animals under clinical suspicion, are analyzed by molecular diagnostic methods [23,24,25].

In terms of surveillance, bulk tank milk (BTM) sampling has been described as a very sensitive, cost-effective, and highly efficient method for the early detection of BTV incursions in farms located in unvaccinated high-risk BTV-free areas [26,27,28]. Bluetongue serogroup-specific antibodies can be detected by a commercial indirect ELISA test based on the recombinant VP7 protein of the virus (ID Screen^®^ Bluetongue Milk Indirect, Innovative Diagnostics, Montpellier, France). The VP7 protein is highly conserved and constitutes an immunodominant serogroup-specific antigen [1]. Moreover, it is the major target for group-specific immunodiagnostics, including all the typical BTV serotypes [29]. The ELISA test has been validated for BTM and individual milk samples, demonstrating a high sensitivity and specificity in both [28,30].

The economical and practical interest in BTM sampling lies in the ease of sample collection, besides the fact that BTM samples are batched. For dairy herds, there is only one sample needed to reveal their sanitary status. In Switzerland, an early detection approach based on a scheme of BTM surveillance was carried out after the BTV-8 incursion in 2007 [27,31]. The first BTM-positive result was observed only one month after the first clinical case had been reported in the country [28]. BTM sampling has also been used to analyze long-lasting immunity after vaccination campaigns, providing information on the immunological status of the herds [27,32]. Recently, BTM surveillance has been employed in the Netherlands after the BTV-3 incursion in September 2023. A retrospective analysis of BTM samples did not reveal the presence of BTV activity in the region [19].

Given the usefulness and versatility of BTM sampling, the availability of standardized reference material of milk samples as controls for diagnostic purposes is essential to harmonize the diagnostic tests among laboratories. Moreover, for a WOAH-listed disease, having updated and fine-tuned surveillance tools with high sensitivity and specificity allowing the detection of BT in low-prevalence herds is crucial, especially in an early infection scenario. The Laboratorio Central de Veterinaria (LCV) based in Algete (Madrid, Spain) is the European Union Reference Laboratory (EURL) for African horse sickness and bluetongue [33]. Within its tasks as EURL are the production and standardization of reference material, as well as the harmonization of laboratory diagnostic methods as a key element for a rapid and homogeneous response to alerts of these diseases [34].

The aim of this study was to generate reference material for performing an ELISA-based serological diagnosis of BT in milk samples. Milk reference material was produced using samples from herds with different BTV statuses. Complementarily, we evaluated the effect of sample pretreatment to increase the analytical sensitivity of the currently available milk ELISA test.

## 2. Materials and Methods

### 2.1. Sampling

The sampling was designed exclusively to obtain well-controlled samples to prepare reference material. Samples were collected to ensure the inclusion of positive farms, which included samples with strong, weak, or medium levels of antibodies, and negative farms.

**Figure 1:**
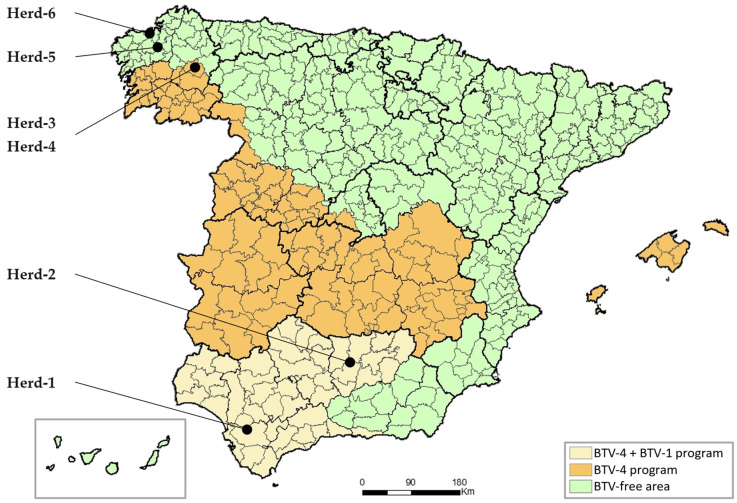
Bluetongue (BT) status in Spain during the sampling according to the National surveillance and control program for BT, and location of herds sampled. Source: Ministry of Agriculture, Fisheries and Food.

According to the National Surveillance Program for the Control and Eradication of Bluetongue 2023 [35], three areas with differential BT status had been established in Spain: two areas under compulsory vaccination program (BTV-4 + BTV-1 program in southern Spain; BTV-4 program in central-northwestern Spain and Balearic Islands) and an unvaccinated BTV-free area (Figure 1).

A total of six dairy herds from Spain from these geographical areas were randomly selected to be sampled between June and July 2023 based on the risk of BTV infection and vaccination status. The sampling included two herds from the BTV-4 + BTV-1 program area, which notified BTV outbreaks in the last two years despite their yearly vaccination program (Herd-1 and Herd-2), two herds from the BTV-4 program area with complete vaccination located in a region where no cases of BTV infection had yet been reported (Herd-3 and Herd-4), and two non-infected and non-vaccinated herds from the BTV-free area (Herd-5 and Herd-6). The geographical location of the herds sampled is shown in Figure 1.

Paired samples of blood (without EDTA) and milk were simultaneously taken from at least 10 lactating cows from each herd, in addition to BTM samples. Due to problems in sample submission, the BTM sample from Herd-2 and one of the individual milk samples from Herd-3 could not be analyzed. The blood samples were incubated at room temperature for 12 h, and the serum fraction was subsequently recovered for analysis. A preservative that contains sodium-azide (Azidiol, Panreac Quimica S.L.U., Castellar del Vallès, Barcelona, Spain) was added to each milk sample in a proportion of 1:1000 *v*/*v* (1 microliter per milliliter of sample). Milk samples were stored for one week at 4 °C and then skimmed by pipetting and stored at –20 °C until testing.

### 2.2. Bluetongue Antibody Detection in Serum and Milk Samples

The serological analyses in serum samples were performed using the blocking ELISA kit INGEZIM BTV Compac 2.0 (Ingenasa, Madrid, Spain). Milk samples were analyzed using the kit ID Screen^®^ Bluetongue Milk Indirect (Innovative Diagnostics, Montpellier, France). Both assays were carried out following the procedures established by the manufacturers. Serum samples were considered positive if their blocking percentage was equal or over the cut-off value set at 40%, and they were considered negative if their blocking percentage was equal or lower than 35%. Samples with blocking percentage values between both cut-off values were considered doubtful. For milk samples, individual milk samples with an S/P% ≤ 90 were considered negative, 90 < S/P% < 110 were considered doubtful, and S/P% ≥ 110 were considered positive. For BTM samples, S/P% values ≤ 30 were considered negative, 30 < S/P% < 40 doubtful, and S/P% ≥ 40 positive.

### 2.3. Milk Reference Material Preparation and Standardization

Once serological assays were performed, individual milk (*n* = 2) and BTM (*n* = 4) samples in different ranges of positivity were selected based on the ELISA results (negative or weak-strong positivity).

Each selected sample was distributed in 25 vials of 0.5 mL and stored at −20 °C. To guarantee the quality of these materials, assigned value, homogeneity, and stability tests were performed, according to an internal procedure based on the quality norm ISO 13528:2022 [36], as detailed below.

#### 2.3.1. Homogeneity Test

The objective of the homogeneity test was to ensure that the items produced were comparable. Three vials of each milk sample were tested by the ELISA in duplicate (*n* = 6). The aliquots prepared from a sample were considered homogeneous if the calculated variability (in S/P%) between aliquots, expressed as between-sample standard deviation (Ss), was less than the check value of tolerated variability of 0.3σ, being “σ” the inter-laboratory variability expected for this type of samples and method:

Homogeneity accepted if Ss < 0.3σ

#### 2.3.2. Assigned Value Test

To assign the qualitative value of each sample, in addition to the homogeneity, two aliquots of each item were analyzed in duplicate by ELISA as described above in two independent assays. To guarantee the variability that may exist between the test conditions, the two assays were performed with different aliquots of each item thawed just before testing, on different days, by a different laboratory technician, and with different pipettes. The assigned qualitative value of each sample was established, accompanied by a quantitative value calculated as the weighted average of the determinations carried out ($\bar{X}$_Av_ ± SD).

#### 2.3.3. Stability Test

Stability tests aimed to demonstrate that the test items are sufficiently stable and will not undergo significant changes during storage and transportation conditions. Two aliquots of each milk sample were thawed and maintained at 4 °C for three months, and two aliquots maintained at room temperature for one week were analyzed by ELISA in duplicate. The preserved aliquots of each sample were considered stable if the difference between the average value obtained in the stability test ($\bar{Y}$) and the expected value from the assigned value tests ($\bar{X}$_Av_) was less than the check value of tolerated variability expected for this type of samples and method (0.3σ):

$$\mathrm{Stability}\;\mathrm{accepted}\;\mathrm{if}\;\left|\overset{-}{X}-\overset{-}{Y}\right|\;<\;0.3\sigma$$

### 2.4. Optimization of Antibody Detection by Milk Protein Concentration Treatment

To improve the sensitivity of the method a procedure to optimize antibody detection in ELISA assays was applied adapting a milk protein precipitation method described in Chaignat et al. [31], with modifications to fine-tune it and increase its performance and efficiency.

Five milliliters of milk samples were subjected to rennet-based casein precipitation by the addition of 100 µL of commercial rennet (Nievi S.L., Erandio, Spain). After 1 h of incubation in a thermostatic bath at 37 °C, samples were centrifuged at 2500× *g* for 5 min, and 4.5 mL of the upper phase (whey fraction) were poured into a conical tube. An equal volume of saturated ammonium sulfate solution (4.1 M) (Merck, Darmstadt, Germany) prepared as described by Wingfield, 1998 [37] was added. After mixing using the vortex, tubes were incubated at room temperature for 30 min in a shaker platform. Then, samples were centrifuged for 20 min at 2500× *g*, supernatants were removed, and pellets were resuspended in 150 µL of wash solution from the ID Screen^®^ Bluetongue Milk Indirect ELISA kit.

To test the procedure, a panel of 7 negative and 17 highly diluted positive milk samples with different levels of positivity were treated in triplicate and tested by the milk ELISA assay (in duplicate) before and after the treatment.

The increase in antibody detection was calculated as % by dividing the average of the three replicates (S/P% values) after treatment by the S/P% result of the corresponding untreated sample.

### 2.5. Statistical Analysis

To compare the serological result of serum, individual milk, and BTM samples between the herds tested, one-way ANOVA followed by Tukey’s multiple comparison test were carried out. For the statistical analysis of the protein concentration procedure globally, a non-parametric Mann–Whitney test was performed. Student’s *t*-test was carried out to assess suitability of concentrated milk samples, by comparison between standard deviation of the milk samples in the duplicated wells of the ELISA test and the samples of concentrated milk protein. For all the analyses, differences were considered significant when the p-value was less than 5% (*p* < 0.05). The statistical software used was Jamovi Stats (V.2.3.13).

## 3. Results

### 3.1. Characterization of Samples

The BTM samples from the infected farms (Herd-1 and Herd-2) tested strongly positive. All individual milk and serum samples were equally positive, except for one cow from Herd-1 (S/P% = 30), which coincided with a low level of BT-antibodies in the serum sample (Blocking% = 42).

In non-infected and vaccinated herds (Herd-3 and Herd-4), all serum samples tested positive without statistically significant differences (*p* = 0.709) compared with the infected herds. However, statistically significant differences were found (*p* < 0.001) when non-infected herds were compared with individual milk samples with lower levels of BTV-specific antibodies. In Herd-3, only three out of nine animals tested positive in individual milk samples, one out of nine tested doubtful, and five out of nine tested negative. The BTM sample tested positive with a lower positivity level than the BTM sample from the infected herds. In Herd-4, none of the individual milk samples tested positive (0/10). However, the BTM sample was positive with a weak level of positivity.

In the herds of the BTV-free area (Herd-5 and Herd-6), all cows tested negative in serum, as well as in individual milk. The BTM samples also tested negative. There were no statistical differences in the S/P% between those herds, but significant differences were observed when compared to both non-infected and vaccinated herds and to the infected herds (*p* < 0.001 in both cases).

The ELISA quantitative values and qualitative results after sampling are summarized in Table 1 Serological results.

**Figure 2:**
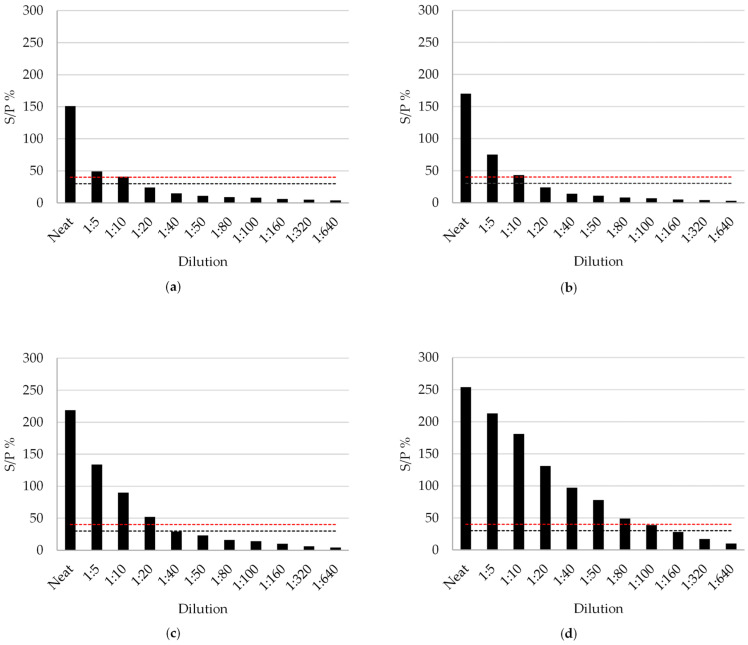
Bulk tank milk sample simulations by several dilutions of individual milk samples with different positivity. (**a**) Milk-1; (**b**) Milk-2; (**c**) Milk-3; (**d**) Milk-4. Results are expressed as S/P%. The upper dashed line (red) indicates the positive cut-off (S/P% ≥ 40), and the lower dashed line (gray) indicates the negative cut-off (S/P% ≤ 30).

Four positive individual milk samples with different positivity values (Milk-1, Milk-2, and Milk-3 from Herd-3; and Milk-4 from Herd-1) were serially diluted in negative milk (S/P% = 5) to simulate BTM samples with different levels of antibodies or different within-herd prevalence (from 20% to 0.15%). Milk-1, Milk-2, and Milk-3 tested positive up to dilution 1:10 to 1:20. Milk-4 tested positive until dilution 1:80 (Figure 2).

### 3.2. Characterization of Milk Reference Material

According to the positivity level of the samples, strong positive samples were selected from the infected herds, weak positive samples from the non-infected and vaccinated herds, and negative samples from herds from the BTV-free area. The identification of each sample, type, origin, and assigned value is summarized in Table 2.

Taking into account the range of positivity and variation obtained in all the analyses carried out in the laboratory with the milk ELISA test (S/P% range: 0–388), the variability expected of the technique (σ) was established in 25% over the maximum quantitative value of 388:

σ=0.25×388=97

According to the established criterion (σ), the samples selected as reference material passed the homogeneity test (Ss < 0.3σ) (Table 3), as well as the stability test ($\left|\overset{-}{X}-\overset{-}{Y}\right|\;<\;0.3\sigma$) (Table 4).

### 3.3. Optimization of Antibody Detection by Milk Protein Concentration Procedure

The panel of 17 positive milk samples was prepared by diluting in BTV antibody-negative milk (S/P% = 2) as explained: 8 out of 17 positive samples were prepared by diluting 1:100 and 1:200 the individual milk samples mentioned in Section 3.1 (Milk-1, Milk-2, Milk-3, and Milk-4), simulating bulk tanks with a within-herd prevalence of 1% and 0.5%, respectively. The last nine samples were prepared for diluting by 1:50, 1:100, and 1:200 three BTM positive samples from Herd-1, Herd-3, and Herd-4, identified as BTM Herd-1, BTM Herd-3, and BTM Herd-4, simulating pools of 50, 100 and 200 BTM samples.

**Figure 3:**
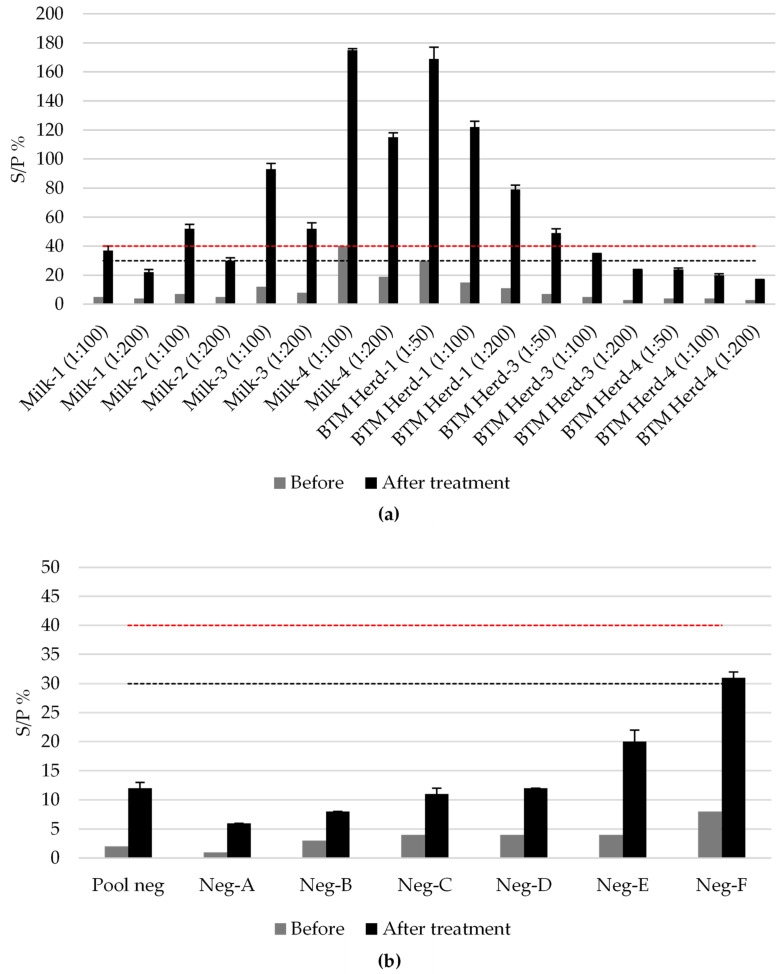
Comparison of antibody detection before and after protein concentration treatment. (**a**) Treatment in diluted positive samples; (**b**) Treatment in negative samples. Error bars show the standard deviation of the replicates after the treatment. Results are expressed as ELISA S/P% value. The upper dashed line (red) indicates the positive cut-off (S/P% ≥ 40), and the lower dashed line (gray) indicates the negative cut-off (S/P% ≤ 30).

All 17 positive milk samples with different levels of positivity were negative before the treatment. Those with an S/P% equal to or higher than 7 in the analysis prior to treatment turned out positive after treatment (Figure 3a). The mean percentage of increase in antibody detection was 650% (confidence interval at 95%: 618–682). Significant statistical differences were observed when comparing ELISA results (in terms of S/P%) before and after the treatment (*p* < 0.001). None of the seven individual negative samples tested positive after treatment, although the negative sample with the highest S/P% reached the limit of the qualitative value assigned as doubtful after treatment (Figure 3b). No significant differences (*p* = 0.241) in the standard deviation of the duplicate wells in the ELISA assays were observed between the milk samples and the concentrated milk protein sample.

## 4. Discussion

The purpose of BT diagnosis in BTM samples is to have a cost-effective and accurate method to indirectly detect the presence of BTV circulation in cattle, even in subclinical animals in unvaccinated BTV-free areas [26,28,30]. Moreover, since BTM samples are routinely taken from dairy herds for milk quality analysis, introducing serological tests in BTM samples for surveillance purposes would be highly practical and economical. In addition, BTM sampling has been demonstrated to be useful in serological tests and also for other relevant animal diseases [38,39,40,41,42,43,44]. However, the provision of standardized and reliable reference materials for the implementation of the method and its harmonization among laboratories is necessary.

Regarding the milk reference materials produced in this study, our quality tests have shown that the milk matrix is reliable for the obtention of homogeneous aliquots in which antibodies remain stable. However, when using similar materials, stability should be evaluated after longer storage periods to establish an adequate expiration date that might vary depending on the period and conditions of storage. Furthermore, when using similar materials, it is worth noting that previous studies confirmed that sodium-azide allows the preservation of milk samples without affecting the results of the ELISA tests for the detection of BTV antibodies [31]. This optimal conservation allows the use of these samples for interlaboratory exchange with minimal expected alteration during transportation or use. In this sense, the reference material produced in this study (Table 2) could be used as additional controls or serve to prepare weak positive controls by dilution for the ELISA test. In this regard, to guarantee the validity of ELISA tests, it is advisable to use an additional weak positive control since weak positive controls, close to the cut-off value, can serve to validate that the test detects weak positive reactions [45].

The sampling was carried out to obtain samples with different levels of positivity. Although a small number of samples have been analyzed in this study, the simultaneous sampling of serum and individual milk from each cow allowed to guarantee the results of the milk analyses. As expected by the sampling design, all herds sampled that had either passed the infection or had been vaccinated showed positive serologies to specific BTV antibodies in serum, as well as positive results in BTM samples. As described by Mars et al. [28], BTM samples provided more sensitivity than individual milk samples with low levels of specific BTV antibodies (see Table 1). Therefore, for individual analysis, serum is the sample of choice, as it provides more sensitivity. Moreover, unlike what was suggested by Chaignat et al. [31], no specificity problems were observed with any milk sample either individual or BTM.

It should be noted that in a previous study, it was suggested that the ELISA value obtained from the BTM sample of a herd could be correlated with the within-herd prevalence [28]. In that study, authors suggested further analysis in several dilutions of BTM samples to accurately establish within-herd prevalences [28]. Based on the analysis of the serial dilution of individual milk, simulating BTM samples with different levels of prevalence (Figure 2), the different positivity of the animals was conducive to high differences in the S/P% value. This leads to the fact that the prevalence estimated through the BTM sample S/P% value could be seriously altered by the influence of a single animal.

In relation to the sensitivity of the ELISA test in pooled BTM samples for BTV surveillance, some limitations have been suggested. At the 2007 EURL Workshop on Bluetongue Diagnostics and Epidemiology (Brussels, Belgium), it was reported that the sensitivity of the milk ELISA test could be compromised when testing over 50 pooled BTM samples or when milk from one positive animal in the early stages of infection is in the bulk tank [46]. The results obtained from analyzing the bulk tank simulations performed in this work with positive samples of different S/P% (Figure 2) are in line with this statement. Only the stronger positive sample diluted at 1:50 proportion (simulating one positive animal in a bulk tank of 50 cows) tested positive (Figure 2d). Moreover, BTM samples diluted at 1:50 simulating pools of 50 BTM did not test positive even in samples from herds with high positivity (Figure 3a, BTM Herd-1 1:50). To solve these shortcomings, a milk protein concentration procedure based on similar methods previously developed for BT [31] and for infectious bovine rhinotracheitis (IBR) [47,48] was evaluated and optimized. Regarding the results from this study (Figure 3), the milk protein concentration treatment produces a high increase in analytical sensitivity, producing positive ELISA results in highly diluted samples with low levels of BTV antibodies (Figure 3a), without false positive induction (Figure 3b). The protein concentration procedure allows for an increase of 650% in the antibody detection in ELISA tests, with repeatable results. In addition, no significant differences in the average standard deviation between ELISA duplicates were observed with the concentrated milk protein samples compared to the milk samples for which the kit is validated, showing that the results of the concentrated milk protein samples are reliable.

The milk protein concentration treatment evaluated in this study is cost-effective; it does not require sophisticated laboratory equipment or expensive reagents, is easy to perform by technicians without specific training, and can be carried out in a working day prior to the ELISA test. On the contrary, it can be laborious to perform with a high number of samples, so its use must be considered in situations where the sensitivity of the assay is of concern. In a surveillance campaign, its usefulness could be restricted to BTM samples with doubtful results in an initial screening test or with suspicion of presenting a low level of BTV antibodies, either due to early infection or because of the dilution effect in a pool of >50 BTM samples. However, it has been tested using a small number of real samples and should be validated before use. Further field trials should be carried out to be applied as a method in a surveillance campaign. Moreover, a commercial kit to concentrate IgG in milk samples has been reported for the detection of anti-IBR antibodies [49,50]. This commercial kit could be an alternative to the treatment described in this study, although it should be evaluated for BT antibody detection.

The results from this study show that highly diluted positive samples that presented an S/P% ≥ 7% tested positive after the protein concentration treatment with the cut-off established by the manufacturer of the milk ELISA test (S/P% ≥ 40). Mars et al. [28] proposed setting lower cut-off values aimed at detecting herds with lower prevalence to increase sensitivity. In that sense, a combination of applying the cut-off assignation suggested by Mars et al. [28], targeted to detect low-prevalence herds, and/or the subsequent application of the milk protein concentration procedure provided here could be considered in an early detection scheme for the surveillance of subclinical BTV infection in a high-risk unvaccinated free area. In agreement, the bulk tank simulations at a prevalence of 1% carried out in this study (Figure 2), would have tested positive by setting a cut-off aimed at detecting this prevalence (S/P% = 6–13 [28]). Furthermore, the presence of antibodies in these samples was subsequently verified by the milk protein concentration procedure with the cut-off established by the manufacturer of the milk ELISA test.

Despite surveillance in milk samples being one of the most effective methods for detecting BTV incursions in an unvaccinated BTV-free region [27,28,30,31], it cannot be used as the exclusive method for surveillance and needs to be combined with precise diagnostic techniques for the detection of the viral agent [23,24]. Moreover, it should not be used in areas where vaccination has been applied since it is not possible to differentiate infected from vaccinated animals. However, due to the fact that it has a high sensitivity in BTM samples, and together with the cost-effectiveness of this sampling method, the milk ELISA employed in this study could be especially useful as a first screening test in an unvaccinated BTV-free area.

## Figures and Tables

**Table 1:** Serological results after sampling. Quantitative values are shown as average of the individual samples ± Standard Deviation of the data.

Herd	Sample (*n*)	ELISA Quantitative Values	ELISA Qualitative Result (Sample Result/*n*)
Herd-1	Blood (10)	90.5 ± 17.08	POS (10/10)
	IM (10)	267.3 ± 91.76	POS (9/10); NEG (1/10)
	BTM (1)	297	POS (1/1)
Herd-2	Blood (10)	96.6 ± 0.97	POS (10/10)
	IM (10)	274.1 ± 40.81	POS (10/10)
	BTM (0)	No sample	No sample
Herd-3	Blood (10)	87.6 ± 7.66	POS (10/10)
	IM (9)	102.6 ± 65.82	POS (3/9) NEG (5/9) DOUBT (1/9)
	BTM (1)	136	POS (1/1)
Herd-4	Blood (10)	82.0 ± 8.94	POS (10/10)
	IM (10)	38.8 ± 20.35	NEG (10/10)
	BTM (1)	56	POS (1/1)
Herd-5	Blood (10)	18.7 ± 3.77	NEG (10/10)
	IM (10)	3.5 ± 2.37	NEG (10/10)
	BTM (1)	4	NEG (1/1)
Herd-6	Blood (10)	19.0 ± 3.83	NEG (10/10)
	IM (10)	2.7 ± 1.16	NEG (10/10)
	BTM (1)	4	NEG (1/1)

IM: individual milk, BTM: bulk tank milk, POS: positive, NEG: negative, DOUBT: doubtful.

**Table 2:** Milk samples selected for reference material and their assigned value.

Identification	Type	Origin	Qualitative Assigned Value (*n*)	S/P% ($\bar{X}$_Av_)	SD
O236	IM	Herd-1	POS (14)	289.4	25.20
O237	IM	Herd-3	POS (14)	167.6	21.52
O238	BTM	Herd-1	POS (14)	261.9	31.20
O239	BTM	Herd-4	POS (14)	72.1	11.08
O240	BTM	Herd-5	NEG (14)	3.2	0.97
O241	BTM	Herd-6	NEG (14)	3.3	0.83

IM: individual milk; BTM: bulk tank milk; $\bar{X}$_Av_ weighted mean value from assigned value assays; SD: standard derivation of the data.

**Table 3:** Homogeneity ELISA test results of the milk reference material.

Identification	Qualitative (*n*)	$S/P\%\;(\bar{X}$_h_)	Ss	0.3σ	Ss < 0.3σ
O236	POS (6)	268.6	0	29	Accepted
O237	POS (6)	152.7	1.75	29	Accepted
O238	POS (6)	241.7	0	29	Accepted
O239	POS (6)	64.2	0	29	Accepted
O240	NEG (6)	2.5	0	29	Accepted
O241	NEG (6)	2.8	0.33	29	Accepted

($\bar{X}$_h_): mean value of homogeneity assays; Ss: between-sample standard deviation; 0.3σ: check value of tolerated variability.

**Table 4:** Stability ELISA test result of the milk reference material stored at 4 °C for three months or at room temperature for one week.

Identification	Stability Conditions	Qualitative (*n*)	$S/P\%\;(\bar{X}$_Av_)	$S/P\%\;(\overset{-}{Y}$)	$\left|\overset{-}{X}–\overset{-}{Y}\right|$	0.3σ	$\left|\overset{-}{X}–\overset{-}{Y}\right|\;<\;0.3$σ
O236	4 °C	POS (4)	289.4	314.8	25.4	29	Accepted
O237		POS (4)	167.6	189.2	21.6	29	Accepted
O238		POS (4)	261.9	278.7	16.8	29	Accepted
O239		POS (4)	72.1	80.4	8.3	29	Accepted
O240		NEG (4)	3.3	2.7	0.6	29	Accepted
O241		NEG (4)	3.3	2.7	0.6	29	Accepted
O236	Room temperature	POS (4)	289.4	314.8	25.4	29	Accepted
O237		POS (4)	167.6	175.5	7.9	29	Accepted
O238		POS (4)	261.9	284.5	22.6	29	Accepted
O239		POS (4)	72.1	81.8	9.7	29	Accepted
O240		NEG (4)	3.2	2.5	0.7	29	Accepted
O241		NEG (4)	3.3	2.8	0.5	29	Accepted

$\bar{X}$_Av_: weighted mean value from assigned value assays; $\overset{-}{Y}$: mean value calculated in the stability test; 0.3σ: check value of tolerated variability.

## Data Availability

The data that support the findings of this study are available from the corresponding author upon reasonable request.
